# Phylogeography Reveals Geographic and Environmental Factors Driving Genetic Differentiation of *Populus* sect. Turanga in Northwest China

**DOI:** 10.3389/fpls.2021.705083

**Published:** 2021-08-11

**Authors:** Zhongshuai Gai, Juntuan Zhai, Xiangxiang Chen, Peipei Jiao, Shanhe Zhang, Jianhao Sun, Rui Qin, Hong Liu, Zhihua Wu, Zhijun Li

**Affiliations:** ^1^Key Laboratory of Biological Resource Protection and Utilization of Tarim Basin, Xinjiang Production and Construction Group, Alar, China; ^2^College of Life Sciences, Tarim University, Alar, China; ^3^Desert Poplar Research Center of Tarim University, Alar, China; ^4^Hubei Provincial Key Laboratory for Protection and Application of Special Plant Germplasm in Wuling Area of China, College of Life Sciences, South-Central University for Nationalities, Wuhan, China

**Keywords:** *Populus euphratica*, *Populus pruinosa*, population structure, genetic diversity, geographical and environmental factors

## Abstract

*Populus* sect. Turanga (hereafter referred to as “*Populus*”), including *Populus euphratica* and *Populus pruinosa*, are the predominant tree species in desert riparian forests in northwestern China. These trees play key roles in maintaining ecosystem balance, curbing desertification, and protecting biodiversity. However, the distribution area of *Populus* forests has been severely diminished and degraded in recent years due to increased habitat destruction and human activity. Understanding the genetic diversity among *Populus* individuals and populations is essential for designing conservation strategies, but comprehensive studies of their genetic diversity in northwest China are lacking. Here, we assessed the population structures and genetic diversity of 1,620 samples from 85 natural populations of *Populus* (59 *P. euphratica* and 26 *P. pruinosa* populations) covering all of northwestern China using 120 single nucleotide polymorphism (SNP) markers. Analysis of population structure revealed significant differentiation between these two sister species and indicated that strong geographical distribution patterns, a geographical barrier, and environmental heterogeneity shaped the extant genetic patterns of *Populus*. Both *P. euphratica* and *P. pruinosa* populations in southern Xinjiang had higher genetic diversity than populations in other clades, perhaps contributing to local geographic structure and strong gene flow. Analysis of molecular variance (AMOVA) identified 15% variance among and 85% variance within subpopulations. Mantel tests suggested that the genetic variation among *P. euphratica* and *P. pruinosa* populations could be explained by both geographical and environmental distance. The genetic diversity of *P. euphratica* showed a significant negative correlation with latitude and longitude and a positive correlation with various environmental factors, such as precipitation of warmest quarter and driest month, temperature seasonality, and annual mean temperature. These findings provide insights into how the genetic differentiation of endangered *Populus* species was driven by geographical and environmental factors, which should be helpful for designing strategies to protect these genetic resources in the future.

## Introduction

*Populus euphratica* Oliv. and *Populus pruinosa* Schrenk are sister species in the section Turanga (*Populus* sect. Turanga, hereafter referred to as “*Populus*”). Both species are predominant trees in the extremely arid desert areas in northwestern China and play important roles in maintaining ecosystem balance and protecting biodiversity (Lang et al., 2015; Zheng et al., 2016). *P. euphratica* and *P. pruinosa* diverged during the Pleistocene era due to climate oscillations and were further isolated by the barrier imposed by the Tianshan Mountains (Wang et al., 2014). Along with the hitchhiking of incidental variations, ancient genetic polymorphisms are thought to have driven the speciation of *P. euphratica* and *P. pruinosa* (Ma et al., 2018). Whereas, *P. pruinosa* is mainly restricted to Xinjiang province in China and the adjacent countries and regions (Wang et al., 1995), the natural distribution areas of *P. euphratica* range from western China and the Middle East to North Africa and southwestern Europe (Ma et al., 2018). Approximately 61% of *P. euphratica* forests worldwide are located in China, with 91.1% of those in Xinjiang province. Alarmingly, the distribution area of *P. euphratica* forests has been seriously diminished or degraded in recent years due to increased habitat destruction and human activity. The protection of *Populus* forests in northwestern China is an extremely urgent issue. One effective strategy for protection of a plant species is to identify priority protection areas or conservation units (Zhang et al., 2020). Assessing the genetic variations in *Populus* populations would be beneficial for the conservation and utilization of the genetic resources of these species and could provide useful basic data on *Populus* germplasm to facilitate breeding.

Studies of genetic diversity and population structure are crucial for exploring natural selection, adaptive evolution, and the genetic relationships of populations within or among *P*. *euphratica* and *P*. *pruinosa*. However, although several molecular population genetics studies have been performed on *P. euphratica* and *P*. *pruinosa*, these studies led to opposite conclusions about their genetic diversity (Wang et al., 2011, 2014; Ma et al., 2018; Zeng et al., 2018). For example, a preliminary investigation using a relatively small number of nuclear and chloroplast DNA markers to investigate the genetic diversity of *P. euphratica* populations (552 *P. euphratica* individuals of 33 natural populations) indicated that this was lower in southern Xinjiang (SX) than in northern Xinjiang (NX) (Zeng et al., 2018). However, whole-genome resequencing of 252 *P. euphratica* individuals from 27 natural populations showed the reverse (Jia et al., 2020). This inconsistency might be attributed to the relatively limited numbers of markers, individuals, or populations studied in narrow geographical areas (Wu et al., 2008; Eusemann et al., 2013; Xu et al., 2013; Wang et al., 2014), or the relatively less advanced methods utilized (e.g., AFLP, RAPD, and SRAP) (Saito et al., 2002; Vonlanthen and Bruelheide, 2010; Wang et al., 2011; Kansu and Kaya, 2020).

The development of molecular markers at the whole-genome level is becoming increasingly accurate and efficient (Verma et al., 2015). Genome-wide single nucleotide polymorphism (SNP) markers are widely used to assess the genotypes and genetic relationships of various populations (Lu et al., 2020; Zhang et al., 2020). Genotyping-in-Thousands by sequencing (GT-seq) has proven to be an efficient SNP genotyping technology (Campbell et al., 2015) and has been widely applied in many fields of research (Bootsma et al., 2020; McKinney et al., 2020; Powell and Campbell, 2020; Schmidt et al., 2020). To better understand the genetic diversity and population differentiation of *P*. *euphratica* and *P*. *pruinosa* to facilitate further conservation and breeding efforts, an analysis of their population genetics using larger-scale samples and more advanced molecular markers is needed.

The natural ranges of *P. euphratica* and *P*. *pruinosa* include different niches that show discontinuous geographical distribution. The barrier represented by the Tianshan Mountains, a major mountain system and biodiversity hotspot, has impeded gene flow between SX and NX in *Populus* populations (Zeng et al., 2018). The geographical differences caused by environmental heterogeneity and climatic fluctuations could drive plant-specific or intraspecific genetic divergence and distribution patterns (Muellner-Riehl, 2019). Geographical distance can also affect the gene flow from one population to another, while environmental factors can influence genetic diversity and help fix a population in a specific ecological niche (Liu et al., 2013). However, there is no report on how the genetic distance and differentiation of *P*. *euphratica* and *P*. *pruinosa* were influenced by geographical and environmental factors. Elucidating the relationship between genetic diversity and geographical or environmental heterogeneity will help explain the discontinuous distribution pattern of *Populus* in northwestern China.

In this study, we collected the leaves of 1,620 accessions from 85 natural populations throughout the natural distribution range of *P. euphratica* (1,183 individuals from 59 natural populations) and *P. pruinosa* (437 individuals from 26 natural populations) in northwest China. Based on our *de novo* assembled reference genome and published resequencing data, we developed 120 high-quality SNPs with MAF ≥ 0.4 for further genetic analysis. Our objectives were to (1) determine the optimal population structures of *P. euphratica* and *P. pruinosa*; (2) evaluate genetic diversity and genetic differentiation between and within the subpopulations; and (3) assess the relationships between population-level genetic diversity and geographical as well as environmental factors. Our findings provide new insights into the genetic differentiation of *P. euphratica* and *P. pruinosa* and lay a genetic foundation for the construction of priority conservation areas of *Populus*.

## Materials and Methods

### Sample Collection and DNA Extraction

Leaves were sampled from 1,183 *P. euphratica* individuals from 59 natural populations in northwest China, including 27 populations from southern Xinjiang (SX), 23 from northern Xinjiang (NX), and 9 total from Gansu (GS), Qinghai (QH), Ningxia (NiX), and Inner Mongolia (NMG) provinces (Supplementary Table 1). Leaves were sampled from 437 *P. pruinosa* individuals from 26 natural populations in Xinjiang province (23 from southern Xinjiang, three from northern Xinjiang), 22 of which co-occurred with *P. euphratica*. To reduce the impact of clonal ramets, all sampled individuals from each population were located at least 100 m apart. The leaves were dried with silica gel in the field. Total DNA was extracted from the leaves and purified using a modified CTAB method (Allen et al., 2006). The integrity of the DNA was evaluated by 0.8% agarose gel electrophoresis, and DNA quality was evaluated based on the *A*_260_/*A*_280_ absorbance ratio of the samples.

### SNP Marker Development

Marker development and sequencing were carried out using the GT-seq method (Campbell et al., 2015). In brief, 120 SNP markers were selected and developed for the *P. euphratica* samples based on our reference genome and resequencing data from *P. euphratica* (Jia et al., 2020). These SNPs, which are uniformly distributed on the 19 *P. euphratica* chromosomes, were retained according to the following criteria (Supplementary Figure 1): (i) SNP interval >50 kb; (ii) minor allele frequency (MAF) ≥0.4; (iii) heterozygosity ≤25%; (iv) missing rates ≤0.25. Primer 3 software (version 2.5.0) was used to design amplification primers for each target site according to the following criteria: (i) the primers ranged from 17 to 32 bases in length; (ii) melting temperature (*T*_m_) values ranged from 60°C to 64°C, with an optimal value of 62°C; (iii) product size was ≤500 bp; (iv) sequenced reads had to be able to cover target sites. Primers for each target site that could specifically amplify the target were obtained (Supplementary Table 2). Libraries were constructed and sequenced (PE150) on the Illumina HiSeq 2500 platform.

### Sequencing Data Analysis

Reliable clean reads were obtained via quality control and filtering of raw reads. Quality control was performed based on strict criteria to remove the following types of reads: (i) adaptor sequences of reads were removed using Cutadapt software (version 1.13); (ii) low-quality bases were removed using Trimmomatic software (Version 0.36); (iii) reads ≤50 bp long were excluded. The reliable clean reads were aligned to the *P. euphratica* reference genome using the BWA-MEM algorithm (version 0.7.15-r1140). After alignment, GATK (version 3.7) (DePristo et al., 2011) and SAMtools (version 1.3.1) (Li and Durbin, 2009) were used for SNP calling. Genotypes at SNP loci were analyzed using an in-house Python script.

### Analysis of Population Structure

Population structure analysis was implemented using STRUCTURE v2.3.4 (Earl and vonHoldt, 2011). The *K*-value was used to evaluate the number of clusters based on the genotyping data; the number of genetic clades was predefined using 10,000 iterations for each run. The optimal *K*-value was determined using Structure Harvester (Earl and vonHoldt, 2011). Principal component analysis (PCA) was carried out using GCTA (Yang et al., 2011). A neighbor-joining (NJ) tree was constructed and visualized using MEGA 7 (Tamura et al., 2013).

### Analysis of Molecular Variance and Genetic Diversity

Analysis of Molecular Variance (AMOVA) was performed and Nei's genetic distance was calculated using GenAlEx 6.503 based on the number of subpopulations (Peakall and Smouse, 2012). Pairwise population differentiation (*F*_ST_) was calculated. Multiple indicators of genetic diversity from each population, such as Shannon's information index (*I*), observed heterozygosity (*H*_o_), expected heterozygosity (*H*_e_), and polymorphism information content (PIC), were obtained using GenAlEx v6.503.

### Analysis of Genetic Isolation

To study the relationships of isolation-by-environment vs. isolation-by-distance based on the genetic differentiation of *P. euphratica* and *P. pruinosa*, 19 bioclimatic variables with a resolution of 2.5 arc-min from the WorldClim Database (http://www.worldclim.org) were obtained (Fick and Hijmans, 2017). Their correlation coefficients were then calculated, and the seven least correlated bioclimatic variables (Spearman's *r* < 0.8) were retained (Supplementary Tables 3, 4). The correlation coefficients between pairwise genetic differentiation and the bioclimatic distance matrix, as well as the geographical distance matrix, were calculated. Mantel tests were performed with 1,000 permutations based on Genepop (https://genepop.curtin.edu.au/) (Rousset, 2008).

To examine the relationships between genetic diversity at the population level and geographical factors, as well as environmental factors according to a previous study (Zhang et al., 2020), the genetic diversity of each population was represented by expected heterozygosity (*H*_e_). Latitude and longitude were considered to be the main geographical factors affecting the *P. euphratica* and *P. pruinosa* populations. All seven retained bioclimatic variables were environmental factors. PCA was used to analyze the climate variables of the sample sites, and the absolute values from standardized PC1 scores were obtained based on correlation analyses using R version 3.5.0.

## Results

### Population Structure and Genetic Relationships

**Figure 1:**
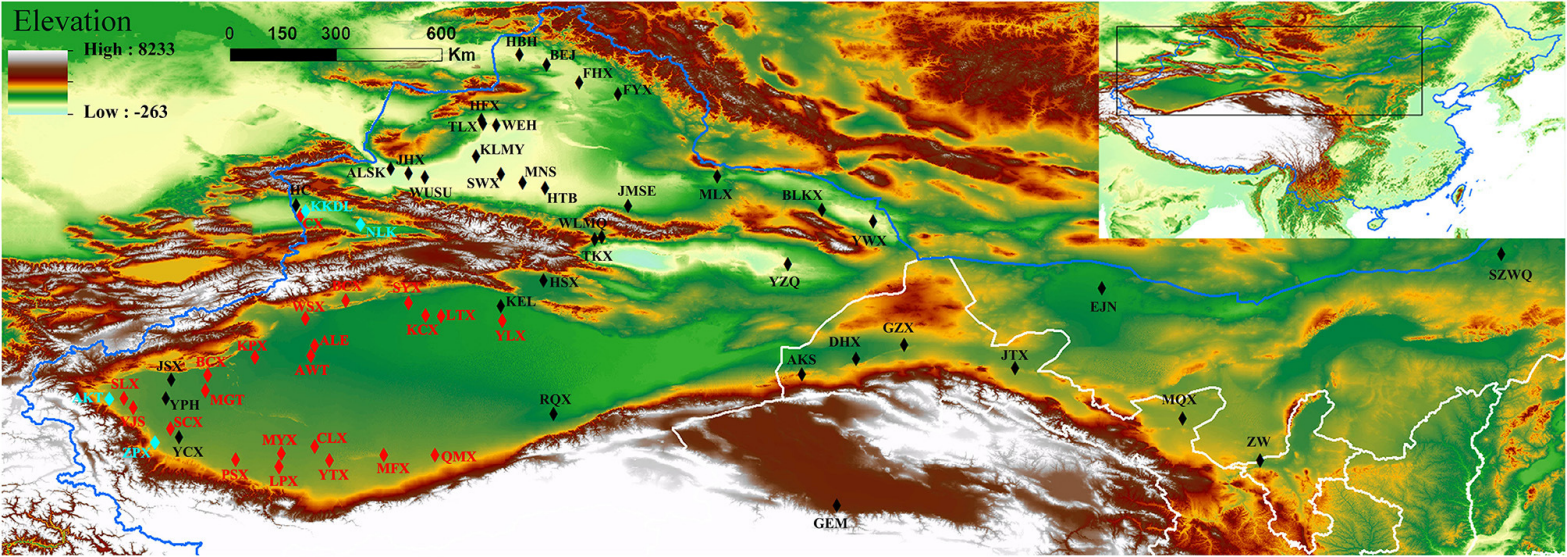
Geographical distributions of the natural populations of *P. euphratica* and *P. pruinosa* analyzed in this study. Different font colors represent the two species: black for *P. euphratica* populations, light blue for *P. pruinosa* populations, and red for overlapping populations. The map was created using the ArcMap package in ArcGIS ver. 10.8 (http://www.esri.com/software/arcgis). The populations are described in Supplementary Table 1.

To comprehensively investigate the genetic diversity of *P. euphratica* (1,183 individuals from 59 natural populations) and *P. pruinosa* (437 individuals from 26 natural populations), we sampled 1,620 individuals from 85 natural *Populus* populations covering the distribution regions throughout northwestern China (Figure 1). The number and distribution area of *P. euphratica* are larger in SX than in NX, and *P. pruinosa* is primarily distributed in SX. The habitat regions of *P. euphratica* showed discontinuous distribution on both banks of inland rivers in the arid regions (e.g., the Tarim River, Yarkant River, and Hotan River of southern Xinjiang and the Ili River, Manas River, and Eerqisi River of northern Xinjiang). In addition, for *P. euphratica*, compared to previous studies, we collected samples from the easternmost population (SZWQ) of China for the first time and added samples from Ningxia Province (ZW). For *P. pruinosa*, compared to previous studies, samples from three populations of *P. pruinosa* (CX, KKDL, and NLKX) were collected for the first time in northern Xinjiang (Figure 1; Supplementary Table 1). In summary, 1,620 samples with high coverage from northwestern China were collected to investigate the genetic diversity and population structures of *P. euphratica* and *P. pruinosa*.

**Figure 2:**
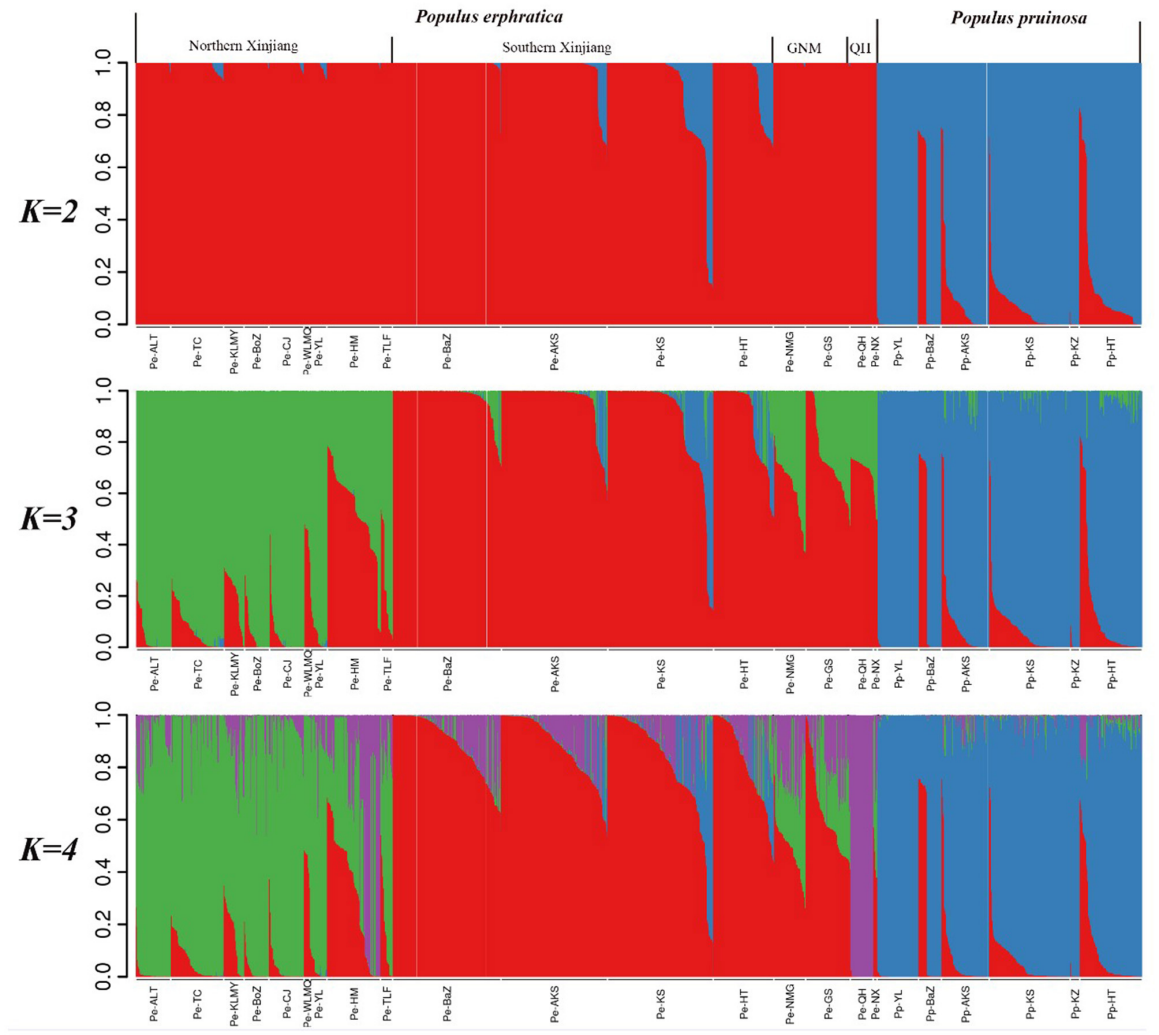
Population stratification for *K* = 2, 3, and 4 determined using STRUCTURE software.

We then carried out population structure analysis based on our genotypic data (Figure 2). When *K* = 2, all individuals were clearly subdivided into two species-specific clusters, highlighting the significant differentiation of *P. euphratica* and *P. pruinosa* at the species level. Notably, a few *P. euphratica* individuals were mixed with the *P. pruinosa* lineage (e.g., MGTX, YPHX, BCX, ALE, YTX), implying the presence of recent gene flow in the two species. When *K* = 3, the *P. euphratica* populations were further divided into southern Xinjiang, northern Xinjiang, and mixed clusters (including GS, NMG, NiX, and QH populations). When *K* = 4, the *P. euphratica* populations were divided into four distinct clades that demonstrated strong geographical distribution patterns (NX, SX, GNM, and QH, with the GNM clade containing individuals from Gansu, Ningxia and Inner Mongolia).

**Figure 3:**
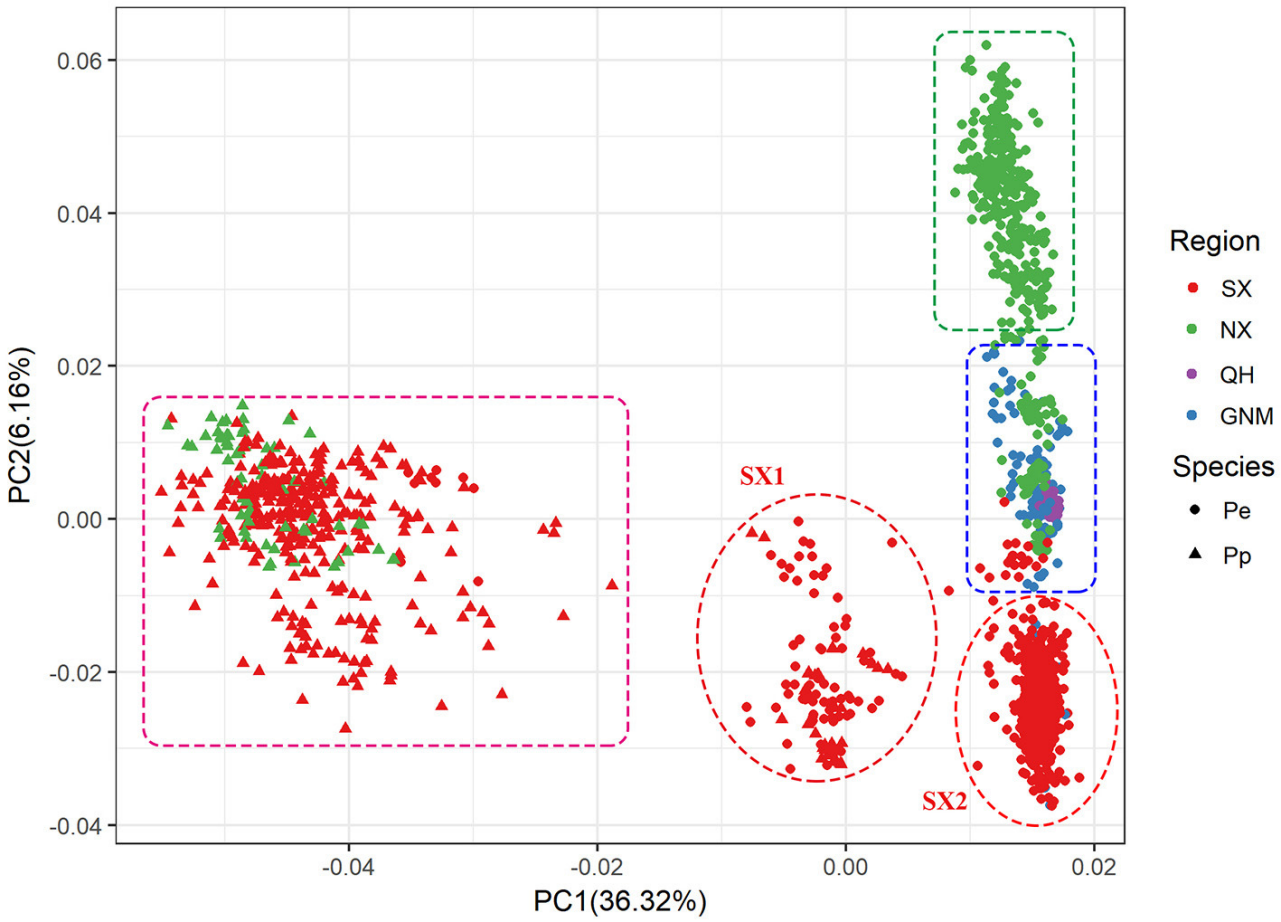
PCA based on genetic distance showing four clustered subpopulations within *P. euphratica* and *P. pruinosa*.

**Figure 4:**
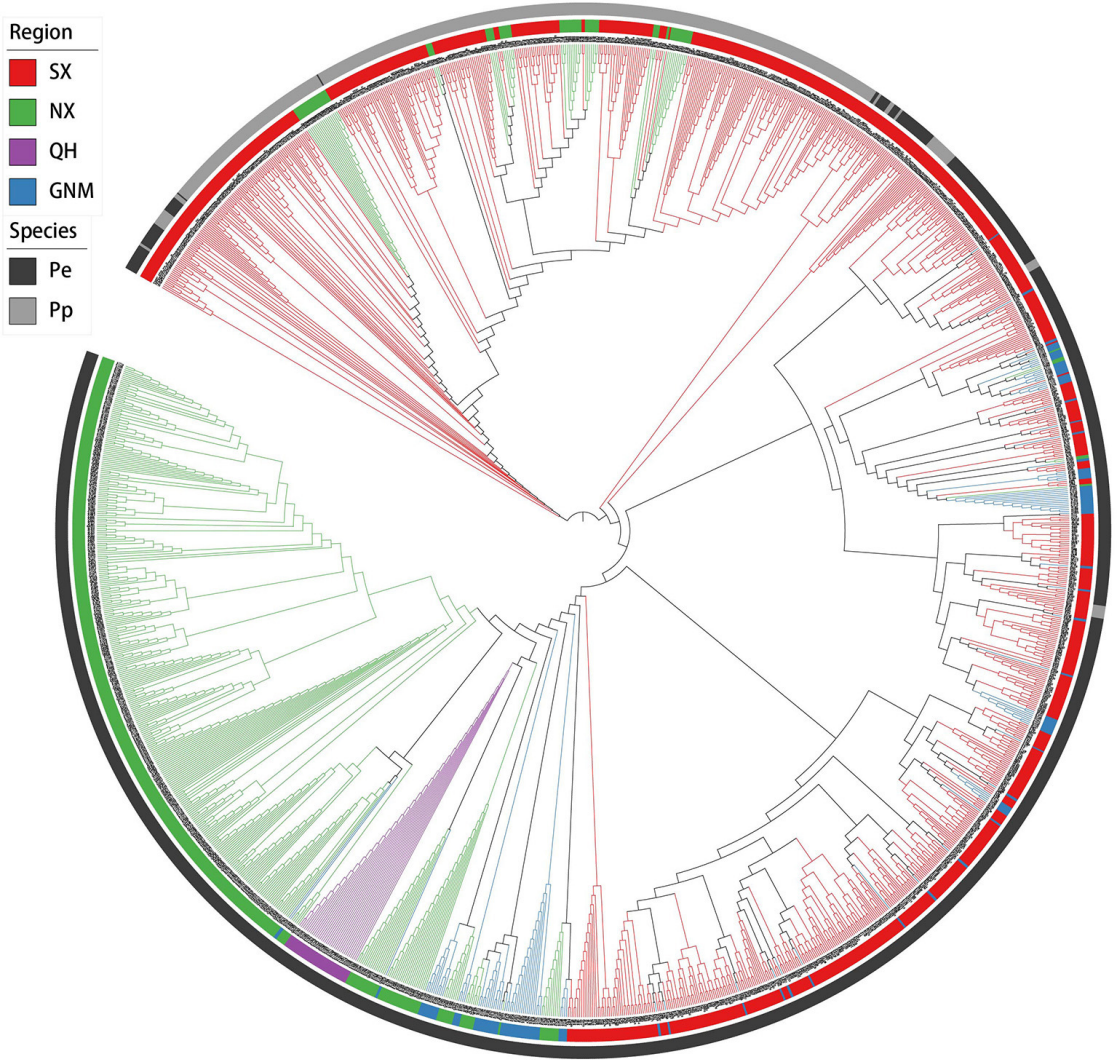
Neighbor-joining phylogenetic tree based on a genetic distance matrix representing the grouping of 1,183 *P. euphratica* and 437 *P. pruinosa* individuals.

PCA and neighbor-joining phylogenetic analysis confirmed our findings about population structure. PC1 mainly explained the variation between *P. pruinosa* and *P. euphratica*, and intermediate individuals (SX1) were primarily from the mixed forests of *P. pruinosa* and *P. euphratica* in southern Xinjiang (e.g., MGTX, ALE, and LPX) (Figure 3; Supplementary Table 5). The presence of these intermediate individuals provides further evidence of gene flow between the two species. PC2 strongly explained the differentiation among the four clades (SX, NX, QH, and GNM) of *P. euphratica*. The QH cluster demonstrated distinct genetic variation compared to the other clades, which might be related to the high altitude (2,709–2,936 m) of this region. The phylogenetic tree, which was constructed to represent the genetic distances between these populations, revealed distinctly different evolutionary relationships among the populations (Figure 4). All three complementary methods (STRUCTURE, PCA, and NJ tree analysis) indicated that distinct genetic differences existed with different geographical distribution patterns at the population level and that the two sister species (*P. euphratica* and *P. pruinosa*) showed significant differentiation at the species level regardless of geographical distance.

### Genetic Diversity of *P. euphratica* and *P. pruinosa* Populations

Based on the population structures of *P. euphratica* and *P. pruinosa* (Figure 2), we calculated the four genetic indices (*H*_e_, *H*_o_, *I*, and PIC) for each clade and population. The genetic diversity values revealed higher diversity for *P. pruinosa* (*H*_e_ = 0.256) than for the four clades of *P. euphratica*. For *P. euphratica*, the SX clade exhibited the highest genetic diversity (*H*_e_ = 0.216), followed by the GNM clade (*H*_e_ = 0.212) and the NX clade (*H*_e_ = 0.206), while the QH clade had the lowest genetic diversity (*H*_e_ = 0.112) (Table 1).

**Table 1:** Comparison of genetic diversity among and within species.

**Pop ID**	***H*_**e**_**	***H*_**o**_**	***I***	**PIC**
Pe GNM	0.212	0.210	0.323	0.171
Pe NX	0.206	0.197	0.317	0.167
Pe QH	0.112	0.205	0.161	0.086
Pe SX	0.216	0.210	0.339	0.176
Pp	0.256	0.194	0.399	0.210

*Pe, P. euphratica; Pp, P. pruinosa; GNM, Gansu, Ningxia, and Inner Mongolia; NX, northern Xinjiang; QH, Qinghai; SX, southern Xinjiang*.

In addition, we calculated the genetic diversity of each population within *P. euphratica* and *P. pruinosa* (Supplementary Tables 6, 7). Among *P. euphratica* populations, the MGTX population (from the KS region of southern Xinjiang) had the highest genetic diversity (*H*_e_ = 0.29) and the TLX population (from the TC region of northern Xinjiang) had the lowest genetic diversity (*H*_e_ = 0.11). On the whole, the populations of southern Xinjiang had relatively high genetic diversity (e.g., MGTX, ALE, YPHX, LPX, PSX, and YTX), followed by one population from Inner Mongolia (EJN) and those from Ningxia (ZW) and Gansu (GZX and JTX), while the populations of northern Xinjiang (e.g., TLX, MLX, FYX, HBHX, and ALSK) and QH (GEM), and the second population from Inner Mongolia (SZWQ), had relatively low genetic diversity (Supplementary Table 6). Among *P. pruinosa* populations, the YTX population (HT region of southern Xinjiang) had the highest genetic diversity (*H*_e_ = 0.28), while the YLX population (BaZ region of southern Xinjiang) had the lowest (*H*_e_ = 0.12). The three *P. pruinosa* populations (CX, KKDL, and NLKX) found only in northern Xinjiang had relatively low genetic diversity (Supplementary Table 7). Overall, the populations of *Populus* in southern Xinjiang had higher genetic diversity than those of other clades. Of these, the MGTX, YTX, ALE, BCX, and KPX populations had relatively high genetic diversity, whereas the KCX, KKDL, AKTX, NLKX, and YLX populations had lower genetic diversity.

### Genetic Differentiation of the Populations

The genetic divergence (*F*_ST_ from 0.118 to 0.160) between *P. euphratica* and *P. pruinosa* was high (Table 2). Of the four clades of *P. euphratica*, we detected high genetic differentiation between QH and GNM (*F*_ST_ = 0.064), moderate differentiation between NX and SX (*F*_ST_ = 0.044) and between NX and QH (*F*_ST_ = 0.044), and lower genetic differentiation between SX and GNM (*F*_ST_ = 0.010).

**Table 2:** Comparison of genetic differentiation among and within species.

**Pop ID**	**Pp**	**NX**	**SX**	**QH**	**GNM**
Pp	0.000				
Pe NX	0.160	0.000			
Pe SX	0.146	0.044	0.000		
Pe QH	0.118	0.042	0.023	0.000	
Pe GNM	0.131	0.022	0.010	0.064	0.000

*Pe, P. euphratica; Pp, P. pruinosa; GNM, Gansu, Ningxia, and Inner Mongolia; NX, northern Xinjiang; QH, Qinghai; SX, southern Xinjiang*.

We further analyzed the genetic differentiation within *P. euphratica* and *P. pruinosa* via pairwise comparisons of different populations. For *P. euphratica*, the genetic differentiation coefficient within populations in southern Xinjiang was low except for MGTX, ALE, and CLX, indicative of strong gene flow among populations. Detailed PCA showed that most of the MGTX (9/11) and ALE (8/11) samples were in SX1 (Supplementary Table 5), suggesting that the higher genetic diversity of MGTX and ALE might have been caused by hybridization of *P. euphratica* and *P. pruinosa*. The genetic differentiation among *P. euphratica* populations in southern Xinjiang and populations of other clades (NX, GNM, and QH) was higher, especially for MLX, FYX, TLX, MQX, SZWQ, and GEM (Supplementary Table 8). However, higher levels of genetic differentiation occurred within the northern Xinjiang population between NX and other populations (GNM and QH). Similar results were obtained regarding the genetic differentiation between the GNM and QH populations. For *P. pruinosa*, the genetic differentiation within populations in southern Xinjiang was high except for MGTX and BCX, and the genetic differentiation among populations in SX and NX was high. Notably, QMX exhibited high genetic differentiation from all other populations of *P. pruinosa* (*F*_ST_ from 0.180 to 0.397), which is associated with the isolated geographic location of this region (Supplementary Table 9). Overall, the populations of *Populus* had relatively high genetic differentiation between SX and NX. AMOVA revealed higher genetic variation within populations (85%, a total of 36% among individuals and 49% within individuals) than among populations (15%) of *P. euphratica*, suggesting a high level of differentiation (Table 3). Nei's genetic distance analysis revealed moderate levels of population differentiation (*F*_ST_ = 0.147) accompanied by a high rate of gene flow between *P. euphratica* populations (*N*_*m*_ = 1.447).

**Table 3:** Analysis of molecular variance of the genetic variation among and within *P. euphratica* populations.

**Source**	**df**	**SS**	**MS**	**Est. Var**.	**%**
Among populations	4	92843.949	23210.987	40.518	15%
Among individuals	1574	526277.637	334.357	99.837	36%
Within individuals	1579	212664.000	134.683	134.683	49%
Total	3157	831785.586		275.038	100%
*F*_ST_	0.147				
*N_*m*_*	1.447				

### Potential Distributions and Genetic Isolation

**Figure 5:**
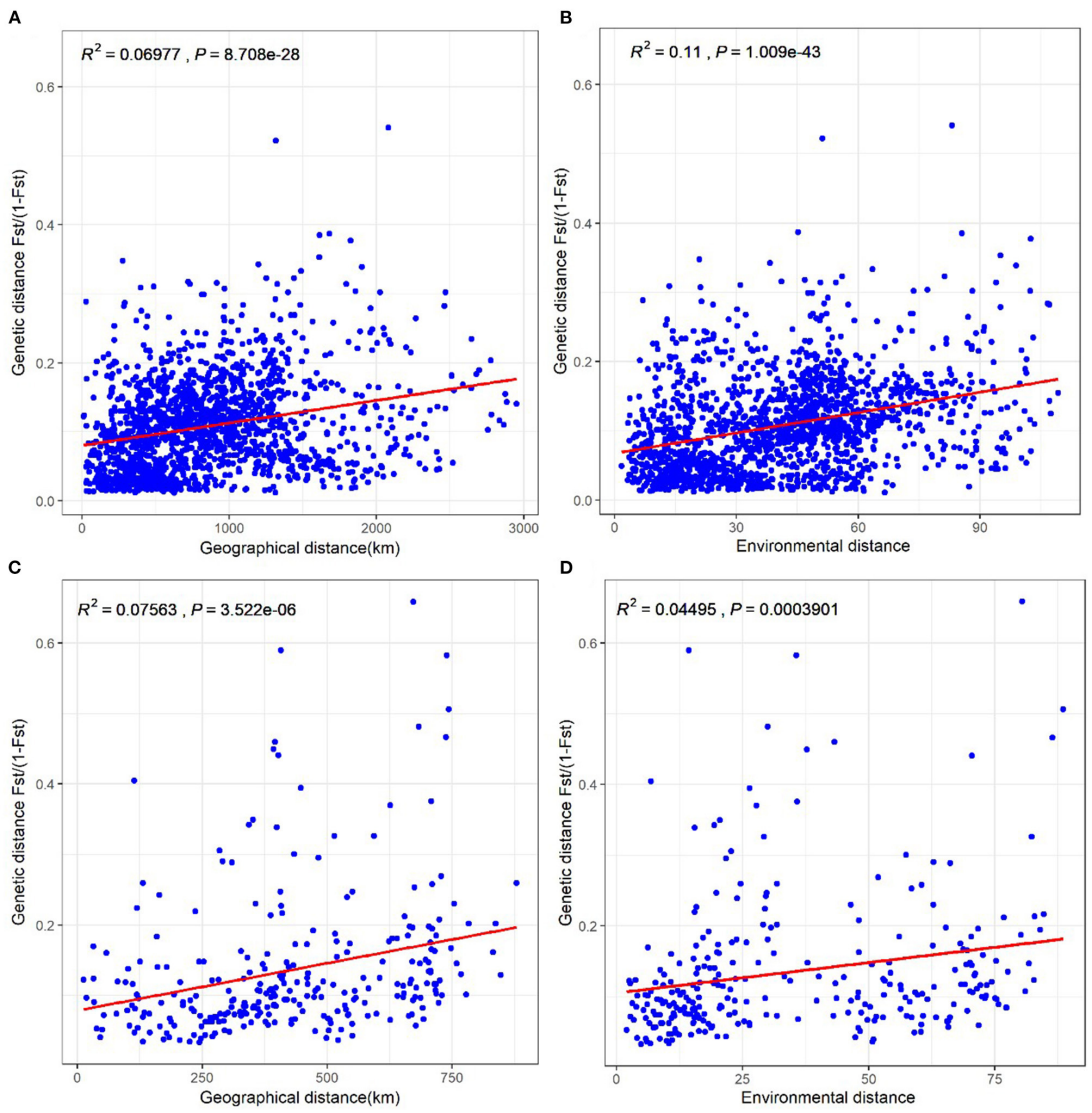
Tests of isolation-by-distance and isolation-by-environment among 58 *P. euphratica* populations **(A,B)** and 24 *P. pruinosa* populations **(C,D)**, respectively. Correlation coefficients were analyzed with data matrixes of genetic distance × geographical distance and genetic distance × environmental distance from pairwise populations.

To determine whether the genetic differentiation in *Populus* is significantly associated with isolation-by-distance and/or isolation-by-environment, we conducted Mantel tests between pairwise genetic differentiation [*F*_ST_/(1 – *F*_ST_)] and a geographical distance matrix, as well as a Euclidean bioclimatic distance matrix with the seven bioclimatic variables. Geographical distance (Figure 5A) and environmental distance (Figure 5B) had significant effects on genetic distance among *P. euphratica* populations. Positive correlations of genetic distance with geographical distance (Figure 5C) and environmental distance (Figure 5D) were also observed for the *P. pruinosa* populations. This finding implies that the genetic differentiation of *Populus* increased as the geographical and environmental distances between populations increased, leading to low gene flow. Also, the different correlations for *P. euphratica* and *P. pruinosa* suggest that environmental distance played a more important role in the genetic differentiation of the *P. euphratica* populations (*R*^2^ = 0.11) than the *P. pruinosa* populations (*R*^2^ = 0.05), while geographical distance contributed equally to the genetic differentiation of both the *P. euphratica* and *P. pruinosa* populations.

**Figure 6:**
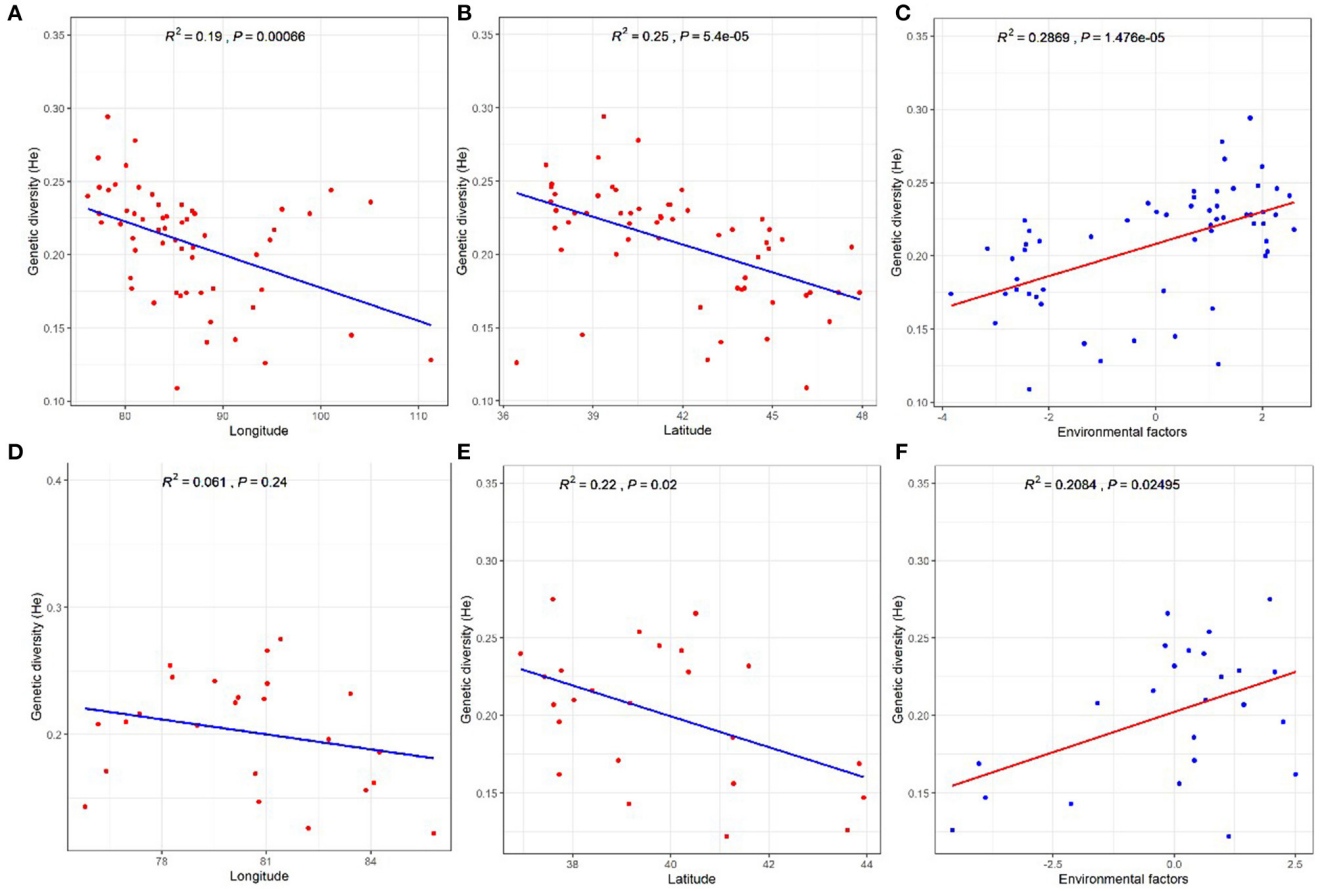
Effects of geographical and environmental factors on genetic diversity in *Populus* populations. **(A–C)** *P. euphratica*. **(D–F)** *P. pruinosa*.

Finally, to test the effects of environmental and geographical factors on the genetic diversity (*H*_e_) of *P. euphratica* and *P. pruinosa* at the population level, we performed correlation analysis between the genetic diversity of *Populus* and latitudinal, longitudinal, and environmental factors. *H*_e_ showed significant negative correlations with longitude and especially latitude for the *P. euphratica* populations (Figures 6A,B), suggesting that the genetic diversity of this species markedly decreased with increasing latitude and longitude. The *H*_e_ of the *P. pruinosa* populations was negatively correlated with latitude but not longitude (Figures 6D,E). Thus, the negative influence of latitude on the genetic diversity of *Populus* was more important than that of longitude. The genetic diversity values of both the *P. euphratica* and *P. pruinosa* populations showed significant positive correlations with environmental factors (Figures 6C,F), suggesting that such factors play important roles in the genetic diversity of both species.

## Discussion

Geographical isolation and environmental heterogeneity are considered to be the crucial drivers of allopatric variance (Evans et al., 2014; Jiang et al., 2018; Binks et al., 2019). A geographic barrier not only facilitates the local adaptation of populations but also can lead to the evolution of populations with unique genetic characteristic (Barnes et al., 2002; Zhang et al., 2020). Our results reveal distinct differentiation between *P. euphratica* populations of the NX and SX clades, suggesting that the Tianshan Mountains may limit gene flow between the two clades and that the differences in local environments and the long period of isolation facilitated their allopatric divergence. It is also thought that the Tianshan Mountains impede seed dispersal and pollen flow in these two regions (Zeng et al., 2018; Jia et al., 2020). Accumulating evidence suggests that climatic fluctuations during the Pleistocene era led to extreme drought and desert expansion in southern Xinjiang (Meng et al., 2015), resulting in habitat fragmentation of desert plant populations. This fragmentation likely affected the geographical distributions of different species, further promoting population divergence and affecting intraspecific genetic diversity (Larmuseau et al., 2009; Ye et al., 2019). Here, Mantel tests showed that the greater the geographical distance, the higher the genetic differentiation and the weaker the gene flow between populations (Figure 5). Moreover, in the current study, we collected CX, KKDL, and NLKX populations of *P. pruinosa* in northern Xinjiang for the first time, and we found that the above analyses were also applicable to *P. pruinosa*.

Genetic diversity refers to the variation of genetic characteristics in a population, which allows the population to adapt to environmental changes. The genetic diversities of each clade were ranked from high to low as follows: SX > GNM > NX > QH. Our results are similar to previous findings based on whole-genome resequencing (Jia et al., 2020) but are not quite consistent with findings based on analysis using 17 nuclear microsatellite loci from *Populus trichocarpa* (Zeng et al., 2018). Perhaps the relatively small number and the *P. trichocarpa* origin of these molecular markers limit their applicability for evaluating genetic diversity in *P. euphratica* and *P. pruinosa*.

Accumulating evidence suggests that gene flow is a creative force in evolution that functions via pollen spread or seed dispersal. Intraspecific genetic admixture can produce new allelic combinations, leading to novel genotypes and phenotypes (Hendry et al., 2002; Olson-Manning et al., 2012; Rius and Darling, 2014). Interestingly, the *P. euphratica* MGTX population showed variable leaf morphology intermediate between that of *P. euphratica* and *P. pruinosa* (Supplementary Figure 2), along with the highest genetic diversity among the *P. euphratica* populations, which could be attributed to gene flow and gene introgression between species (Supplementary Table 5). In addition, *P. euphratica* and *P. pruinosa* are dioecious, with males flowering earlier than females in each species, although the flowering periods basically overlap. However, *P. euphratica* flowers slightly earlier than *P. pruinosa*, suggesting that asymmetrical gene flow from male *P. pruinosa* to female *P. euphratica* might increase the probability of genetic variation (Wang et al., 1995, 2011). This observation likely explains why the co-occurring population of *P. euphratica* and *P. pruinosa* had higher genetic diversity than the other populations of each species.

Current assumptions suggest that palaeogeographical changes (Wang et al., 2013) or heterogeneous environments (Forester et al., 2016) might also affect the spatial genetic patterns of plants. We found here that both geographical distance and environmental heterogeneity significantly influenced the genetic variation of *P. euphratica* and *P. pruinosa*, suggesting that the genetic distance within the isolates increased with geographical distance. Changes in longitude and latitude represent the effects of geographical distance on genetic variation, which further strengthens our hypothesis that the presence of a geographical barrier facilitated the genetic differentiation of these populations. Local environmental differences and a long period of isolation facilitate allopatric divergence. For example, the environmental factors of NMG, NiX, and GS were intermediate between those of SX and NX (Jia et al., 2020), which might have contributed to the formation of the GNM clade. Meanwhile, analysis of the effects of geographical and environmental factors on the genetic diversity of *P. euphratica* and *P. pruinosa* showed that genetic diversity decreased with increasing latitude and longitude. In particular, the genetic diversity of *Populus* is more closely related to latitude, which might be related to differences in photoperiod or temperature in different latitudes. This notion requires further study.

A recent study of environmental factors influencing the geographical distribution of *P. euphratica* showed that this is affected by precipitation of the driest month and the warmest quarter, the soil moisture content (10–40 cm underground), soil moisture around the root system, and the evapotranspiration of soil water (Guo et al., 2020). Precipitation of the driest month and the warmest quarter is an important source of soil groundwater recharge during the growing season of *P. euphratica*. Soil water evaporation limits the effective recharge of soil groundwater, and soil water content and root soil moisture are important indices of effective soil groundwater recharge. Notably, precipitation of the driest month and the warmest month was opportunely encountered flooding season in southern Xinjiang (Zheng et al., 2016; Gai et al., 2020). In general, the peak of yearly seed release (at the end of July) coincides with the annual flooding period in that region (Eusemann et al., 2013). These favorable conditions may be conducive to the reproduction of *P. euphratica* and *P. pruinosa*, perhaps explaining why southern Xinjiang owns the largest *P. euphratica* and *P. pruinosa* forests in northwest China.

The high genetic diversity of the SX populations may result from its distinct local geographic structure and strong gene flow (Shen et al., 2014). Southern Xinjiang represents the widest distribution area of *P. euphratica* globally. The high genetic diversity of *P. euphratica* populations in this region reflects the large effective population size and large amount of endemic gene resources. To better protect these genetic *Populus* resources, more effort should focus on the intensity of flood diversion and irrigation and the protection of *P. euphratica* forests in southern Xinjiang, regardless of their ecological significance or importance to genetic conservation. More attention should also be paid to the replacement and renewal of *P. euphratica* forests in northern Xinjiang. Finally, the dramatic decreases in *P. pruinosa* populations underline the need to preserve this species, which is important not only in itself but as a precious genetic resource to enrich the gene pool of *P. euphratica*. Our genetic data here indicate that a comprehensive strategy is needed to maximize the protection of desert poplar.

## Conclusion

In this study, we explored population structure and genetic diversity among 1,620 samples covering the full distribution ranges of *P. euphratica* and *P. pruinosa* populations in northwest China. Our study found that the natural populations of *P. euphratica* in northwest China show strong geographical distribution patterns and that *P. euphratica* and *P. pruinosa* populations in southern Xinjiang have higher genetic diversity than the populations of other clades. Mantel tests suggested that both geographical and environmental distance significantly influence genetic variation in *P. euphratica* and *P. pruinosa*: the genetic diversity of *P. euphratica* markedly decreases with increasing latitude and longitude and is positively correlated with environmental factors. Finally, the higher genetic diversity in southern Xinjiang may contribute to the local geographic structure and strong gene flow in these populations.

## Data Availability Statement

The datasets presented in this study can be found in online repositories. The reference genome of Populus euphratica is available at our Populus genome database (http://118.24.202.236:20003/). The names of the SNP repository and accession number(s) can be found below: https://www.ncbi.nlm.nih.gov/, PRJNA738601.

## Author Contributions

ZL and ZW conceived and designed the study and provided suggestions and comments about the manuscript. ZG collected and analyzed the data and wrote the manuscript. JZ, XC, PJ, SZ, and JS collected the samples. RQ and HL provided constructive comments. ZW revised the manuscript. All authors read and approved the manuscript.

## Conflict of Interest

The authors declare that the research was conducted in the absence of any commercial or financial relationships that could be construed as a potential conflict of interest.

## Publisher's Note

All claims expressed in this article are solely those of the authors and do not necessarily represent those of their affiliated organizations, or those of the publisher, the editors and the reviewers. Any product that may be evaluated in this article, or claim that may be made by its manufacturer, is not guaranteed or endorsed by the publisher.

## Supplementary Material

Supplementary Table 1 Overview of information about the samples used in this study.

Supplementary Table 2 The primers used for each target SNP site.

Supplementary Table 3 Spearman's rank correlation coefficients (*r*) for the seven remaining bioclimatic variables in *P. euphratica*.

Supplementary Table 4 Spearman's rank correlation coefficients (*r*) for the seven remaining bioclimatic variables in *P. pruinosa*.

Supplementary Table 5 Information about the SX1 samples analyzed by PCA.

Supplementary Table 6 Genetic diversity analysis of *P. euphratica* samples from 59 natural populations.

Supplementary Table 7 Genetic diversity analysis of *P. pruinosa* samples from 26 natural populations.

Supplementary Table 8 Genetic differentiation coefficients among *P. euphratica* populations.

Supplementary Table 9 Genetic differentiation coefficients among *P. pruinosa* populations.

Supplementary Figure 1 Distribution of selected SNPs on the 19 *P. euphratica* chromosomes.

Supplementary Figure 2 Phenotypic variation in the leaves of *P. euphratica* and *P. pruinosa* natural populations in Maigaiti County, southern Xinjiang.
