# Supplementary figures and images for: Phylogeography Reveals Geographic and Environmental Factors Driving Genetic Differentiation of Populus sect. Turanga in Northwest China
Source: Front Plant Sci. 2021 Aug 11;12:705083. doi: 10.3389/fpls.2021.705083 (PMC8385373; doi:10.3389/fpls.2021.705083)

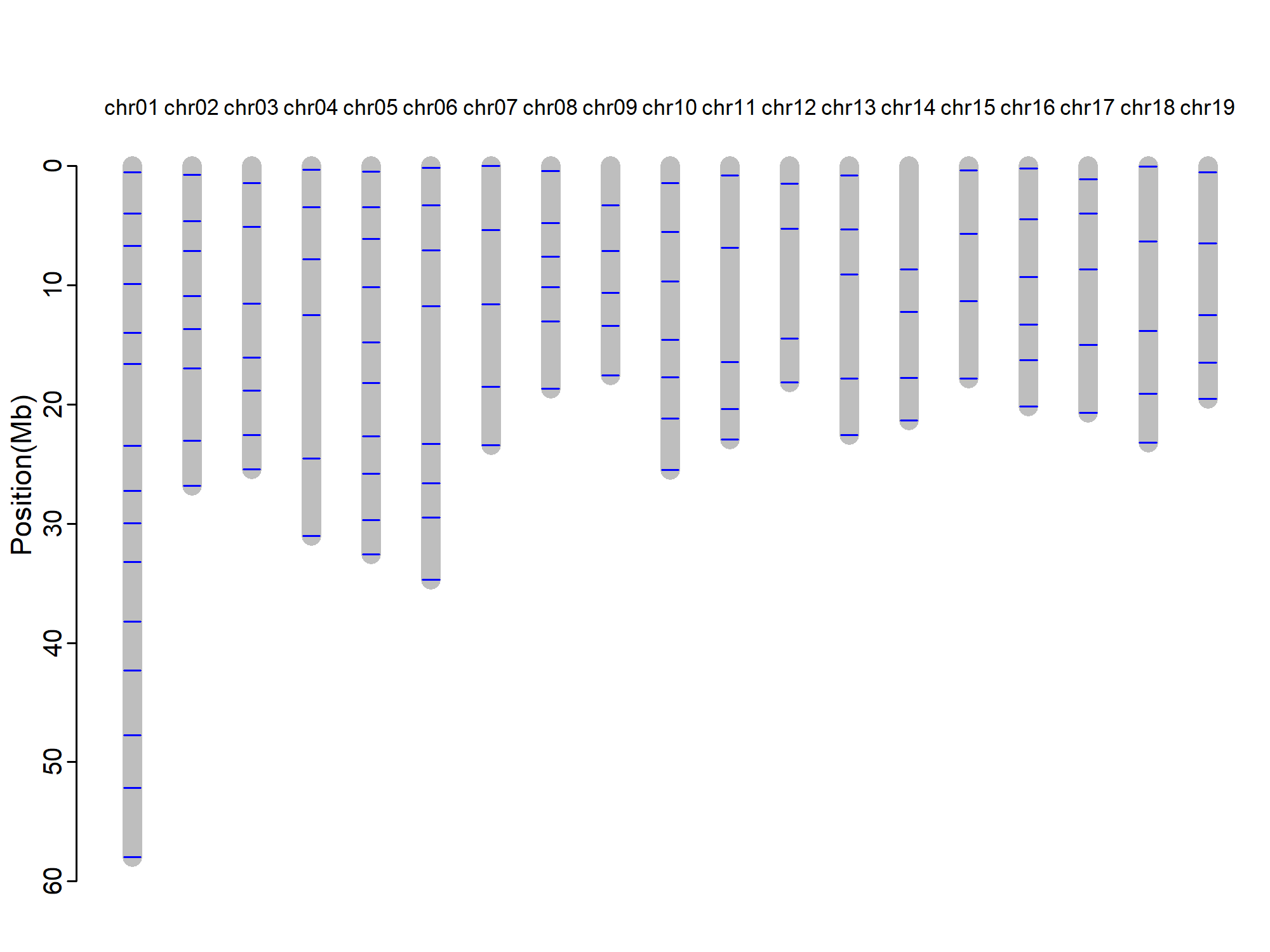

Supplement: Supplementary Figure 1 — Distribution of selected SNPs on the 19 P. euphratica chromosomes. [file Image_1.PNG]
